# Lead, cadmium, and other trace elements in the liver of golden eagles and white-tailed eagles: recent data from Poland and a systematic review of previous studies

**DOI:** 10.1007/s11356-022-25024-y

**Published:** 2022-12-30

**Authors:** Maciej Marcin Durkalec, Agnieszka Nawrocka, Ignacy Kitowski, Aleksandra Filipek, Bartosz Sell, Mirosława Kmiecik, Piotr Jedziniak

**Affiliations:** 1 Department of Pharmacology and Toxicology, National Veterinary Research Institute, Aleja Partyzantów 57, 24-100 Puławy, Poland; 2 Department of Zoology and Animal Ecology, University of Life Sciences in Lublin, Akademicka 13, 20-950 Lublin, Poland

**Keywords:** Biomonitoring, Eagles, Exposure, Metalloids, Trace metals, Raptors, Lead, Cadmium

## Abstract

The golden eagle (*Aquila chrysaetos*) and the white-tailed eagle (*Haliaeetus albicilla*), being apex predators and facultative scavengers, can bioaccumulate different environmental contaminants, including toxic elements that may adversely affect their health. We analyzed the levels of cadmium (Cd), lead (Pb), and other metals and metalloids, including arsenic (As), barium (Ba), beryllium (Be), cobalt (Co), chromium (Cr), copper (Cu), iron (Fe), magnesium (Mg), manganese (Mn), molybdenum (Mo), selenium (Se), thorium (Th), thallium (Tl), uranium (U), vanadium (V), and zinc (Zn) in liver samples taken from three golden eagles and 36 white-tailed eagles that were found dead across Poland to verify their exposure. We also used a systematic review to summarize the available literature data on Cd, Pb, and other studied elements in the liver of both eagle species. Analyses of trace elements in the liver samples of the Polish eagles revealed interspecific differences in Cd, Cu, and Mn and differences in Co, Mn, Tl, and Zn among study regions. All elements tested except Pb were below the suggested thresholds linked with adverse health effects in birds. The hepatic Pb found in almost half of all the tested individuals suggests environmental exposure to this toxic element. One of the tested white-tailed eagles had hepatic Pb above the threshold of sublethal poisoning. Although our results seem optimistic, as previous Polish studies showed a higher prevalence of birds with hepatic Pb exceeding the toxicity threshold, they indicate that exposure to this toxic metal could still pose an additional threat to the health of Polish eagles.

**Graphical abstract:**

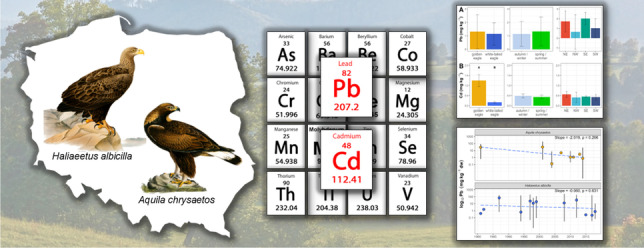

**Supplementary Information:**

The online version contains supplementary material available at 10.1007/s11356-022-25024-y.

## Introduction

The golden eagle (*Aquila chrysaetos*) and white-tailed eagle or white-tailed sea-eagle (*Haliaeetus albicilla*) are two of the largest birds of prey that are considered keystone species in many countries, including Poland (Kitowski et al. 2017). The populations of these two large raptors have dramatically declined in the nineteenth and twentieth centuries, mainly due to persecution since they were considered a threat to livestock and game animals (Nebel et al. 2015). As a result of many efforts made toward the conservation of the eagles, the number of individuals of both species in Europe have increased in the last decades, and the conservation status of their European populations has been changed from endangered to least concern (LC) (BirdLife International 2021). The golden eagle is very rare in Poland and is distributed mainly in the southern, mountainous part of the country. Its population was 34 breeding pairs in 2020 (Wardecki et al. 2021). The white-tailed eagle is far more abundant across entire country. It has been estimated that the current number of breeding pairs in Poland may be around 1000–1400 (Eagle Conservation Committee 2011). A recent report on the monitoring program of the population of birds in Poland showed a twofold increase in white-tailed eagle abundance over the past decades (Wardecki et al. 2021). Despite ongoing efforts to protect eagles, there are still several threats that can negatively affect their conservation, including habitat loss, intentional killing, intentional or unintentional poisoning, electrocution, collisions with wind turbines, climate change, and exposure to environmental pollutants including toxic elements (Katzner et al. 2012; Isomursu et al. 2018; McClure et al. 2018). Lead (Pb) is one of the many environmental pollutants that can adversely affect the health of eagles and deserves the most attention as it is found to be a common cause of poisoning in birds of prey (Williams et al. 2017), primarily due to exposure caused by scavenging carrion contaminated by remnants of Pb-based hunting ammunition (Pain et al. 2019). A recent review summarizing the effects of Pb on birds of prey revealed that the golden eagle and white-tailed eagle, as facultative scavengers, are among the species of raptors most likely to suffer from Pb poisoning, which can be seen in reports from many countries (Monclús et al. 2020). Previous reports from Poland also highlighted a high prevalence of liver Pb above the toxicity threshold in white-tailed eagle (Kalisińska et al. 2006; Kitowski et al. 2017). Cadmium (Cd) is another toxic metal that has no biological function in higher organisms. It can enter the body through respiratory and dietary routes. For birds, dietary is considered the main route of Cd exposure (Wayland and Scheuhammer 2011). Once absorbed, Cd accumulates mainly in the liver and kidneys, which account for 67–97% of the total body burden of this metal in bird’s organism (Wayland and Scheuhammer 2011). Experimental studies on birds showed numerous adverse effects of Cd exposure such as nephrotoxicity, reproductive disorders, disruption of nutrient intake and metabolism, endocrine disruption, and behavioral alterations (Wayland and Scheuhammer 2011). Cadmium has significant potential for bioaccumulation, and its concentration in subsequent trophic levels shows a U-shaped curve with high concentrations in low trophic levels groups including plants and herbivores, low concentrations in organisms on intermediate trophic levels, and finally, increased concentrations in top-level predators (Burger 2008). Eagles, as apex predators, are considered suitable biomonitors of trace element environmental pollution that can provide early warning of the potential impacts of these contaminants on humans (Gómez-Ramírez et al. 2014; Badry et al. 2019).

Our study aimed to (1) quantify the concentrations of Cd and Pb and other metals and metalloids (As, Ba, Be, Co, Cr, Cu, Fe, Mg, Mn, Mo, Se, Th, Tl, U, V, and Zn) in liver samples of golden eagles and white-tailed eagles collected in Poland; (2) verify the influence of species, season, and country site on hepatic trace elements in eagles; (3) assess the exposure of the tested individuals to potentially toxic elements; (4) summarize the existing data of metals and metalloids in the liver of both species studied; and (5) verify potential temporal trends in the levels of Cd and Pb in both species using our results and data reported elsewhere.

## Materials and methods

### Sampling

**Fig. 1:**
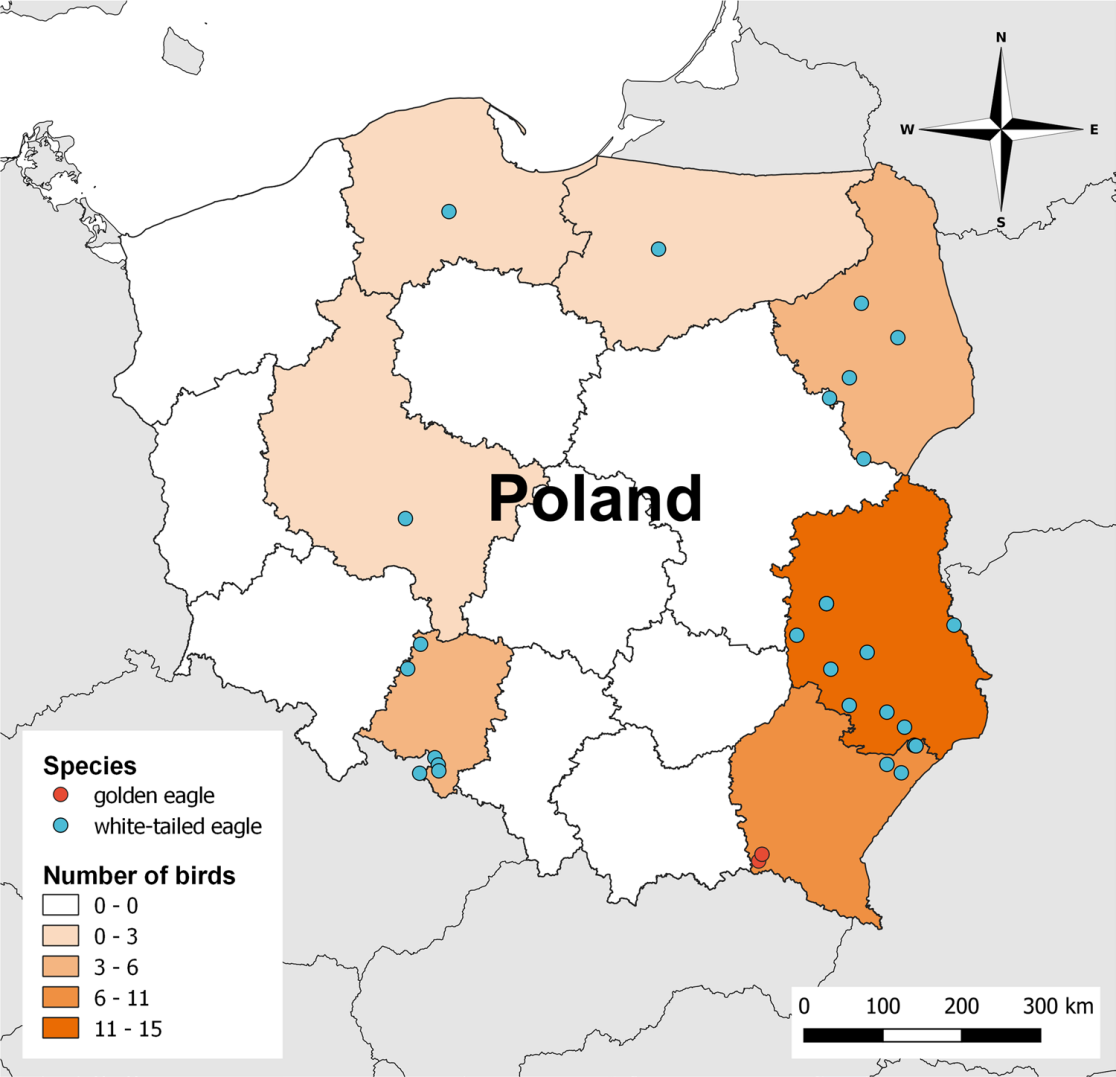
Map of Poland with sampling locations and study areas. The dots represent the coordinates of where the dead eagles were found. The number of birds found in the province is shown on a color scale if exact coordinates of the location were unavailable. The map was created using QGIS software version 3.22 (QGIS.org 2022)

All samples of liver tissue used in this study were collected from golden eagle (*Aquila chrysaetos*) (*N* = 3) and white-tailed eagle (*Haliaeetus albicilla*) (*N* = 36) individuals that were found dead across different regions of Poland between 2010 and 2020 (Fig. 1). The sampling was performed by people dealing with raptor conservation (veterinarians, employees of the National State Forests, National Parks, and wildlife rescue centers). Collected liver samples were packed separately in labeled safe sampling bags or containers, frozen immediately, and sent to the Department of Pharmacology and Toxicology of the National Veterinary Research Institute in Puławy for toxicological diagnostics targeting residues of rodenticides and pesticides that are illegally used for pest control (Sell et al. 2022). When possible, the information about the birds, including species, date of sampling, and location, was recorded by the sampler on the spot. Unfortunately, detailed information on the age, sex, body weight, and biometric measurements was unavailable for some of the birds examined in this study; thus, we decided not to include those factors in our analysis.

### Elemental analysis

The concentrations of metals and metalloids, including As, Ba, Be, Cd, Co, Cr, Cu, Fe, Mg, Mn, Mo, Pb, Se, Th, Tl, U, V, and Zn, in the liver samples were determined using a validated and accredited method (Accreditation No. AB485) in the laboratory of the Department of Pharmacology and Toxicology which is certified according to PN-EN ISO/IEC 17025:2018–02 and serves as a National Reference Laboratory for Heavy Metals in Food of Animal Origin and Feed. Briefly, homogenized liver samples were mineralized in a mixture of 3 mL concentrated nitric acid HNO_3_ (69%, Normatom, VWR, Leuven, Belgium) and 0.5 mL of 30% non-stabilized hydrogen peroxide solution (Suprapur, Merck, Darmstadt, Germany) using a Speedwave 4 microwave digestion system equipped with DAC-100 vessels (Berghof, Einingen, Germany). The analysis of the total element content was performed by ICP-MS (7700x, Agilent, Tokyo, Japan) as described previously (Durkalec et al. 2018). The reliability of the obtained results was verified by analyzing the DORM-4 fish protein certified reference material (NRC, Ontario, Canada) (Supplementary Table S1). The method’s performance was also confirmed by satisfactory results of Proficiency Tests that were organized by the European Reference Laboratories (EURL-MN, Technical University of Denmark (DTU), Lyngby, Denmark. The moisture content of the homogenized liver samples was determined with a halogen moisture analyzer (HR83, Mettler Toledo, Greifensee, Switzerland) using a standard drying program recommended by the manufacturer. The obtained moisture was used to convert the trace element results from wet weight (ww) to dry weight (dw). All results given in the article are expressed as mg kg^−1^ dw.

### Exposure assessment

To interpret the obtained levels of potentially toxic elements in the livers of the eagles, we used published suggested thresholds linked with sublethal adverse effects in various birds (Supplementary Table S2). The following thresholds were used to interpret hepatic Pb levels: 6.6 mg kg^−1^ dw, which may indicate subclinical poisoning, and 33 mg kg^−1^ dw linked with severe clinical poisoning (Franson and Pain 2011). We also used a level of 1 mg kg^−1^ dw that was suggested as abnormal (Neumann 2009) which may indicate extended exposure to Pb from various sources. For the interpretation of hepatic Cd, we adopted the following thresholds: 3 mg kg^−1^ which indicates increased exposure of birds (Scheuhammer 1987), and 148.5 mg kg^−1^ dw, which corresponds to 45 mg kg^−1^ ww and indicates hepatotoxicity, nephrotoxicity, and testicular toxicity (Wayland and Scheuhammer 2011). Because no clear toxicity threshold for hepatic As in birds was available, we used a value of 1.3 mg kg^−1^, which has been linked with various adverse health effects in mallard (*Anas platyrhynchos*) ducklings exposed to sodium arsenate through their diet (Camardese et al. 1990). For the hepatic levels of other trace elements, including Cu (Puls 1988), Mn (Puls 1988), Se (Eisler 1993; Ohlendorf and Heinz 2011), and Zn (Puls 1988; Eisler 1993) which are essential for birds but can induce toxic effects if ingested in excess, we adopted the levels suggested for poultry and wild species of birds (Supplementary Table S2).

### Systematic literature search

We used a systematic review to summarize the available literature data on specific trace elements in the liver of golden eagles and white-tailed eagles worldwide. When performing the systematic review, we followed the RepOrting standards for Systematic Evidence Syntheses (ROSES) statement (Haddaway et al. 2018). A literature search was made during the last 2 weeks of April 2022 using three different search engines: PubMed (www.pubmed.ncbi.nlm.nih.gov), Scopus (www.scopus.com), and Web of Science (www.webofscience.com). We searched for available articles without a specific timeframe, including the title, abstract, and keywords. For searching, we used the following combination of terms: ((white-tailed AND eagle*) OR (*Haliaeetus* AND *albicilla*) OR (golden AND eagle*) OR (*Aquila* AND *chrysaetos*)) AND (“toxic element*” OR “trace element*” OR “heavy metal*” OR “toxic metal*” OR “arsenic” OR “cadmium” OR “cobalt” OR “chromium” OR “copper” OR “iron” OR “magnesium” OR “manganese” OR “molybdenum” OR “lead” OR “selenium” OR “thallium” OR “uranium” OR “vanadium” OR “zinc”)). We also screened reference lists of the reviews for trace elements in raptors to identify other relevant publications by hand. The first selection of studies was based on an initial title and abstract screening, and the second step involved reviewing the full text of the article for eligibility (Supplementary Fig. S1). We included publications that met the pre-established criteria: (1) they reported results of peer-reviewed studies; (2) they contained extractable raw or statistical summary data of specific trace elements in the liver of golden eagle or white-tailed eagle; (3) they described the analytical method used; (4) they were published in English. We excluded the following types of articles: review articles, experimental trials, articles reporting only the number of birds with liver element level within a particular range (prevalence study), case reports not providing a precise concentration of the element, and articles available only as an abstract. Data extraction from the eligible publications was performed by one author (MD) using a predefined Excel form (Supplementary Dataset). The following information was extracted from each study: author’s name, year of publication, study timeframe, journal country, region, number of birds sampled, element tested, value (concentration of the specific element), value type (arithmetic mean, geometric mean, median or singe result), minimum (min), maximum (max), result expression (dry weight, dw, or wet weight, ww), analytical technique used, if the article describes quality control of analyses (QC), type of QC samples used, journal name, article type, bibliographic data, DOI. For those articles that reported the results only graphically (Ecke et al. 2017; Slabe et al. 2022), a WebPlotDigitizer was used to estimate central values from graphs (Burda et al. 2017).

### Data treatment and statistical analysis

#### Analysis of obtained data

Prior to analysis, data below the LOQs were replaced with half of the LOQ of the specific relevant element. The Shapiro–Wilk test was used to check the normality of the data distribution (Yap and Sim 2011). Because the distribution of our data was different than normal, and the normality was also not achieved by log-transformation, non-parametric methods were applied. We used generalized linear models (GLMs) with Gaussian family and the log-link function to verify the effect of the following explanatory variables on the concentrations of specific elements in the liver of eagles: species (golden eagle, *N* = 3; white-tailed eagle, *N* = 36), region (NE Poland, *N* = 6; NW Poland, *N* = 2; SE Poland, *N* = 25; SW Poland, *N* = 6), and season (fall–winter, *N* = 17; spring–summer, *N* = 22). Individual birds were categorized by season according to the date of carcass finding and time intervals of meteorological seasons (fall–winter from 1 of September to 28 of February; spring–summer from 1 of March to 31 of August). Wald’s chi-square test implemented in the regTermTest command from the *survey* package version 4.1–1 (Lumley 2004) was used to verify the significance of the explanatory variables within the model. Then, we calculated estimated marginal means and verified the differences in concentrations of the elements between certain factors by pairwise comparison with *mvt* adjustment using the *emmeans* package version 1.7.0 (Lenth 2021). The Spearman’s rank correlation coefficient was used to verify the relationships between specific elements in the liver of the white-tailed eagle. The golden eagle was excluded from the correlation analysis due to the small sample size. The statistical analysis was performed using R version 4.1.3 (R Core Team 2022) with R-Studio (RStudio Team 2022). The *dplyr* package (Wickham et al. 2022) was used for data manipulation and calculation of the descriptive statistics, including the mean, standard deviation (SD), median, and interquartile range (IQR). The results were visualized using the *ggplot2* package version 3.3.5 (Wickham 2016), and correlations between specific elements were plotted using the *corrplot* package version 0.92 (Wei and Simko 2021).

#### Analysis of literature data

We needed to preprocess the extracted data before the analysis so the average concentration of the element, the concentration range, and the number of birds can be visualized in a comparable manner. For studies that reported raw data of individual birds (Falandysz et al. 1988; Iwata et al. 2000), we calculated the arithmetic mean, min, and max from the original results. For those publications that reported results only by specific categories (Ecke et al. 2017; Kitowski et al. 2017; Viner and Kagan 2021; Helander et al. 2021), we calculated the weighted mean based on central values and the number of birds of each category. When needed, original data were converted from ww to dw, using a conversion factor of 3.3 (Helander et al. 2021). The extracted and unified literature data were then used to assess the temporal trends in hepatic Pb and Cd in golden eagles and white-tailed eagles. For this purpose, we used the middle of the study period and the averaged concentrations of the element (weighted mean of the study results conducted during the period). The Ljung-Box test was used for testing data autocorrelation (Ljung and Box 1978) and the Mann–Kendall trend test implemented in the *trend* package version 1.1.4 was used to verify the presence of trends in the concentrations of the elements in the livers of the eagles (Pohlert 2020). The results were summarized graphically using the *ggplot2* package version 3.3.5 (Wickham 2016).

## Results and discussion

In total, we quantified 15 elements, excluding Be, Th, and U, that were below the limits of quantification of our method and for which the percentage of left-censored data were 87%, 90%, and 100%, respectively. The descriptive statistics of the observed concentrations of metals and metalloids in the livers of the golden and white-tailed eagles are summarized in Supplementary Table S3. The raw results are available in the supplementary Excel file (Supplementary Dataset). A total of 367 studies were found using three search engines and the predefined queries, and one was retrieved by hand searching, of which 204 remained after duplicate removal. After the initial screening, 107 studies were excluded based on title, which yielded 97 articles that appeared to be eligible for full-text review. The assessment of the available full-text articles allowed us to exclude 71 articles for specific reasons (Supplementary Fig. S1) and identified 27 studies that met our predefined criteria and contained results of trace element analyses in the liver of eagles that could be extracted. Of these, ten studies concern golden eagles, 14 white-tailed eagles, and three studies were focused on both species. A total of 14 articles dealt with Pb only, six described the results of three toxic elements, including Pb, and seven described the results of various elements, including toxic and essential ones. Inductively coupled plasma mass spectrometry (ICP-MS) was the most frequently used technique (15 of 27 studies), followed by atomic absorption spectroscopy (AAS, 8 of 27 studies), inductively coupled plasma atomic emission spectroscopy (ICP-AES, 3 of 27 studies), and in one study that was conducted over an extended period, two different analytical techniques (AAS and ICP-MS) were used. In three articles, the authors did not state which technique was used to determine the elemental content, and eight articles lacked a description of the quality control of the analyses. All information extracted from the selected studies are available in the supplementary Excel file (Supplementary Dataset).

### Lead

**Fig. 2:**
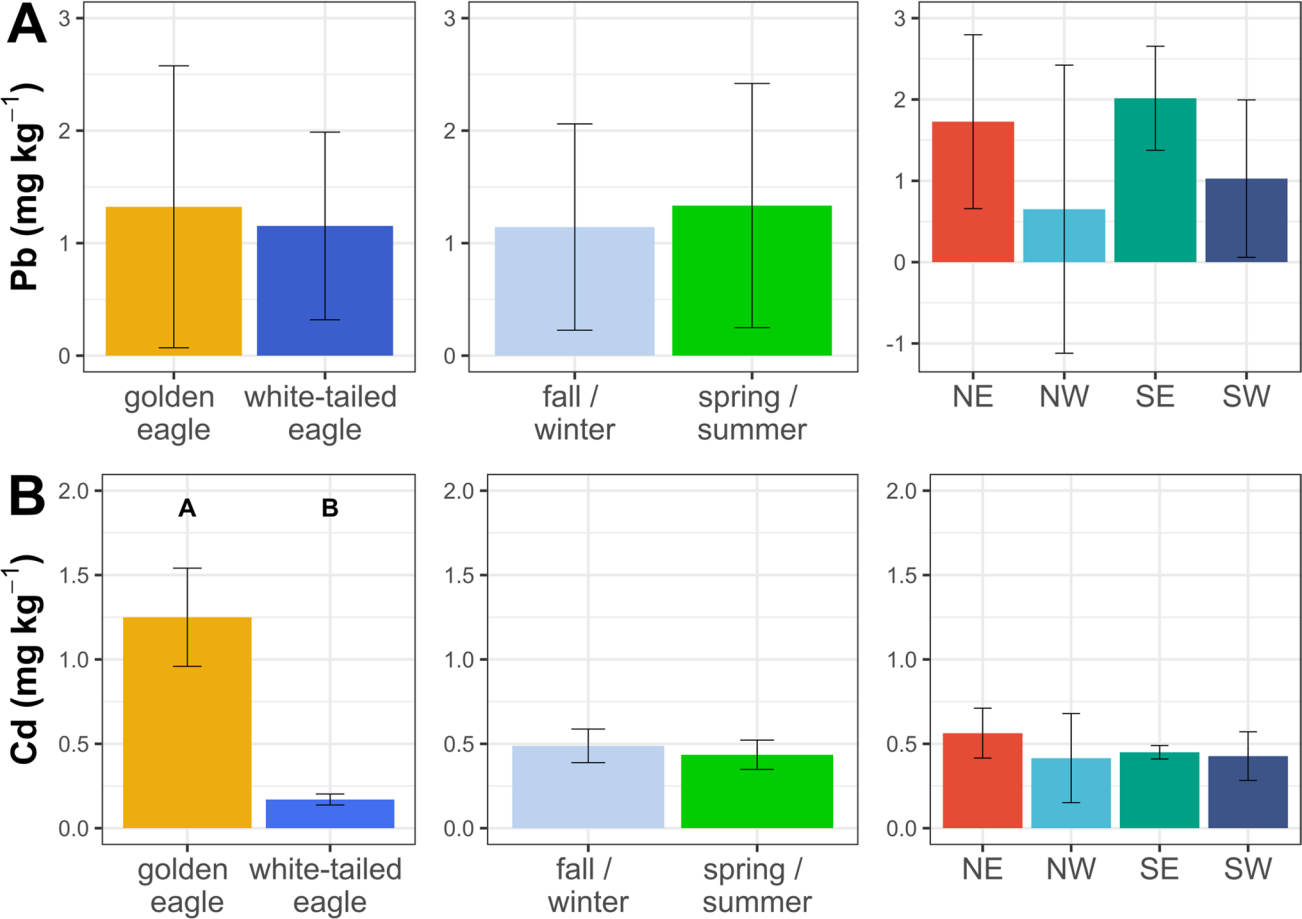
Differences in concentrations of Pb (**A**) and Cd (**B**) in the livers between golden eagles and white-tailed eagles, fall–winter and spring–summer seasons, and among regions of Poland (in mg kg^−1^ dw). Bar and whisker plots show estimated marginal means and standard errors that were computed from the GLM model and back-transformed from the log scale. Differences between marginal means were verified on the log scale (*p* ≤ 0.05)

**Fig. 3:**
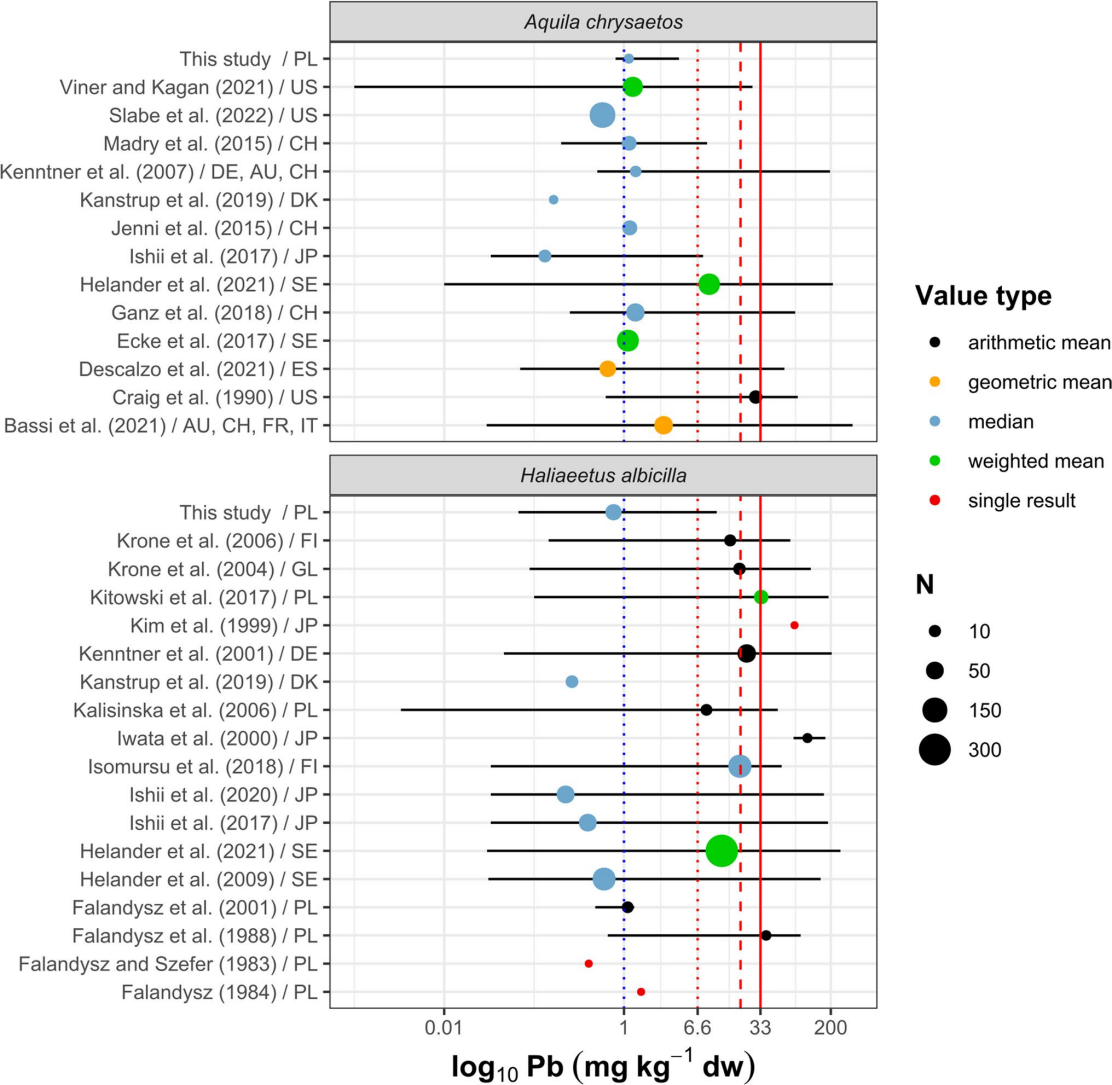
Concentrations of Pb in the liver of golden eagles (*Aquila chrysaetos*) and white-tailed eagles (*Haliaeetus albicilla*). Dots represent central value (mean or median), and whiskers show minimum and maximum. The dot size represents the number of birds tested (*N*). Gray indicates results that were converted from ww to dw; black, original values; and red, results obtained in this study. Vertical lines represent literature threshold; blue dotted line, abnormal Pb levels in the liver of eagles (Neumann 2009); red dotted line, subclinical poisoning (Franson and Pain 2011); red dashed line, clinical poisoning; and red solid line, severe clinical poisoning (Franson and Pain 2011). Countries are indicated by a two-letter ISO 3166–1 alpha-2 code. (For interpretation of the references to color in this figure legend, the reader is referred to the web version of this article.) (Falandysz and Szefer 1983; Falandysz 1984; Falandysz et al. 1988, 2001; Craig et al. 1990; Kim et al. 1999; Iwata et al. 2000; Kenntner et al. 2001, 2007; Krone et al. 2004, 2006; Kalisińska et al. 2006; Helander et al. 2021, 2009; Jenni et al. 2015; Madry et al. 2015; Ishii et al. 2017, 2020; Ecke et al. 2017; Isomursu et al. 2018; Ganz et al. 2018; Kanstrup et al. 2019; Bassi et al. 2021; Descalzo et al. 2021; Viner and Kagan 2021; Slabe et al. 2022)

**Fig. 4:**
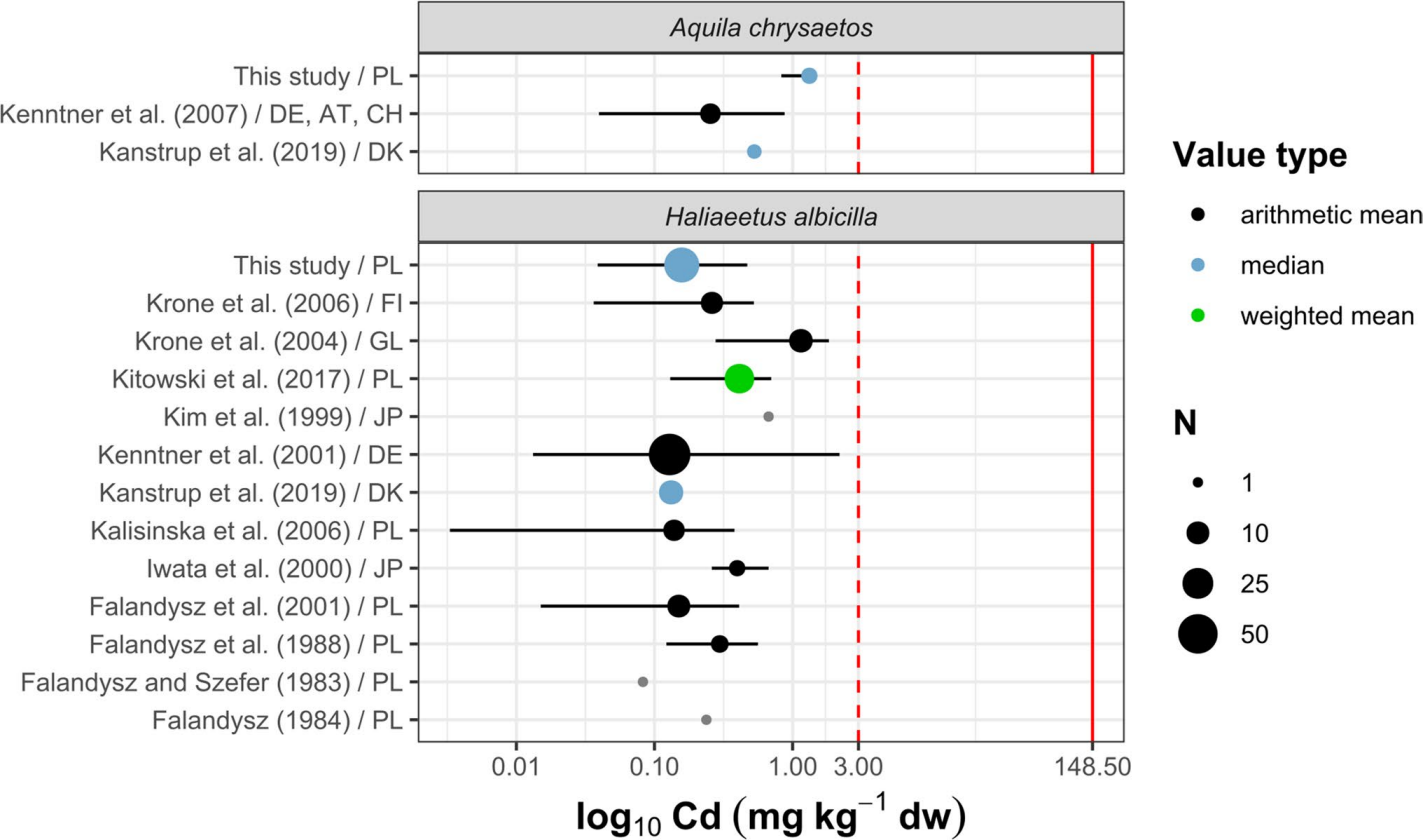
Concentrations of Cd in the livers of golden eagles (*Aquila chrysaetos*) and white-tailed eagles (*Haliaeetus albicilla*) (in mg kg^−1^ dw). Dots represent central value (mean or median), and whiskers show minimum and maximum. The dot size represents the number of birds tested (*N*). Gray indicates results that were converted from ww to dw; black, original values; and red, results obtained in this study. The red dashed line represents level of 3.0 mg kg^−1^ indicating increased exposure of birds to Cd (Scheuhammer 1987); the red solid line represents the threshold of hepatic, renal, or testicular toxicity (> 148.5 mg kg^−1^) (Wayland and Scheuhammer 2011). Countries are indicated using a two-letter ISO 3166–1 alpha-2 code. (For interpretation of the references to color in this figure legend, the reader is referred to the web version of this article.) (Falandysz and Szefer 1983; Falandysz 1984; Falandysz et al. 1988, 2001; Kim et al. 1999; Iwata et al. 2000; Kenntner et al. 2001, 2007; Krone et al. 2004, 2006; Kalisińska et al. 2006; Kitowski et al. 2017; Kanstrup et al. 2019)

Lead has no biological role in living organisms and shows a wide range of adverse effects on physiological and biochemical systems in birds’ organisms, including the cardiovascular, nervous, renal, hematopoietic, immune, and reproductive systems (ATSDR 2007). The hepatic Pb in the studied eagles did not show any interspecific (Wald’s *X*^2^ = 0.05, *p* = 0.83), seasonal (Wald’s *X*^2^ = 0.14, *p* = 0.71), or site-specific differences (Wald’s *X*^2^ = 0.72, *p* = 0.87) (Fig. 2B). The concentration of Pb in the livers of the golden eagles found in our study was comparable to those reported in other parts of the world excluding the values by Bassi et al. (2021) from the alpine regions of Austria, France, Italy, and Switzerland, where the concentration of this metal was more than twice as high (Fig. 3). Golden eagles found in different areas of Sweden also had liver Pb about eight-fold higher than reported in this study (Helander et al. 2021) (Fig. 4). The level of Pb in the white-tailed eagles’ livers in this report was comparable with the concentrations found in northern Poland in the 1980s (Falandysz and Szefer 1983; Falandysz 1984; Falandysz et al. 2001) but 11-fold lower compared to those reported from white-tailed eagles found in the northern part of Poland in the late 1990s (Kalisińska et al. 2006). Our results were also more than 40-times lower compared to those found in white-tailed eagles between 2009 and 2014 in the eastern part of the country, where 36% of tested individuals had liver Pb above the sublethal toxicity threshold, and about 32% of them exceeded the level of clinical poisoning (Kitowski et al. 2017). The authors concluded that the high Pb concentrations in the birds’ livers were due to their exposure to Pb from feeding on Pb-contaminated game carcasses, which often comprise their diet during the winter half of the year when fish are difficult to prey on. Other studies confirmed a higher frequency of game mammals and birds in the diet of the white-tailed eagle during the August–March period, which corresponds to the hunting season in Europe (Nadjafzadeh et al. 2013, 2016). Lead-based ammunition is considered one of the most important sources of Pb exposure for raptors, especially during the fall–winter season, when driven hunts are held. Based on a questionnaire conducted among hunters and an examination of sampled bullets used for moose hunting in Fennoscandia, it was estimated that, on average, between 10 and 26% of the bullet may remain in the carcass (Stokke et al. 2017). Of this, about 30% can remain in the gut pile and offal, which is often left in the field after the carcass is eviscerated (Stokke et al. 2017). A radiographic analysis of white-tailed eagles’ regurgitated pellets showed the birds’ increased exposure to Pb during fall and winter (14.3% of the regurgitated pellets with metal residues from September to February vs. 7.6% from March to August) (Menzel and Krone 2021). It is also worth mentioning that eagles are selective hunters and quickly analyze the behavior of their prey, making them prone to go after injured animals that are easier to chase, including individuals wounded by shots (Helander et al. 2021). Our results showed that only one of the tested white-tailed eagles had hepatic Pb more than one and a half times higher than the suggested threshold of 6.6 mg kg^−1^, which indicates subclinical poisoning. However, 46% of the total number of birds tested (44% of white-tailed eagles (16/36) and two golden eagles) had Pb levels higher than that indicating Pb exposure (1 mg kg^−1^). Although only one white-tailed eagle in our study has hepatic Pb higher than the subclinical poisoning threshold, the median Pb concentration in this species (Supplementary Table S3) was about two-fold higher than reported in white-tailed eagles from Hokkaido (Japan) (Ishii et al. 2017, 2020) and Denmark (Kanstrup et al. 2019), where Pb-based ammunition has been banned. A survey of pheasant and mallard carcasses conducted in 2016–2018 in Denmark, where the regulation prohibiting the use of Pb-based ammunition for hunting and clay shooting was introduced in 1996, showed that only 2% of birds were shot with Pb-based ammunition (Kanstrup and Balsby 2019). The effectiveness of the ban was also confirmed in the USA, where a significant decrease in the percentage of individuals with high blood lead levels has been observed in many waterfowl species, primarily ducks (Anderson et al. 2000; Samuel and Bowers 2000). A study conducted at Doñana National Park in southern Spain also showed that significant decrease in the number of Spanish imperial eagles (*Aquila adalberti*) with ingested lead shots was observed after a ban on waterfowl hunting in the area (Mateo et al. 2007). The EU’s recent restriction on the use of lead shot in wetlands throughout the EU, which will take effect in February 2023, should reduce waterbird species’ exposure to this toxic element (EC 2021). Therefore, raptors that prey on waterbirds, including white-tailed eagles, are expected to have lower exposure to Pb. Because the regulation covers only wetlands, golden eagles and other raptors that prefer terrestrial habitats may still be exposed to Pb residues by scavenging gut piles or unrecovered carcasses of game animals that were shot using Pb-based rifle ammunition (Krone 2018; Pain et al. 2019). We believe switching to lead-free ammunition seems to be the most effective solution to reduce the risk of Pb exposure for eagles and other scavenging animals. Although lead-free hunting ammunition exists on the market, its use is still negligible mainly due to different factors that affect hunters’ attitudes, including socioeconomic ones, poor availability of lead-free ammunition, concerns about its efficacy and safety, and the habit of using conventional ammunition (Thomas et al. 2015, 2016; Schulz et al. 2021). Therefore, there is a need for education on the effects of spent Pb-based ammunition and a constructive discussion that considers the interests of hunting organizations, ammunition manufacturers, and legislators so a consensus can be developed and joint action taken to reduce Pb emissions into the environment.

### Cadmium

The levels of Cd in birds depend on their trophic level, feeding habits, ecosystem, age, and physiological state (Wayland and Scheuhammer 2011). The GLM analysis confirmed interspecific differences in hepatic Cd (Wald’s *X*^2^ = 165.02, *p* < 2.2 10^−16^). The estimated marginal mean concentration of Cd in the liver of golden eagles was 1.25 mg kg^−1^ and was 7.4 times higher than that found in the liver of white-tailed eagles. We did not find any seasonal (Wald’s *X*^2^ = 1.04, *p* = 0.31) or site-specific differences (Wald’s *X*^2^ = 0.72, *p* = 0.87) in hepatic Cd in either species (Fig. 2A). We hypothesize that this difference may be due to the different dietary preferences of both species. White-tailed eagles generally prey on fish and waterfowl, and mammals comprise only a few percent of their diet (Zawadzka 1999; Ekblad et al. 2020). A study on the feeding habits of raptors from Wigry National Park in NE Poland showed that the biomass of prey of white-tailed eagles consisted mainly of birds (70%), including different species of the Anatidae family (19.1%) and Eurasian coot (*Fulica atra*), and several small to medium-sized fish (26.8%), with common bream (*Abramis brama*) as the dominant species (10.4%) (Zawadzka 1999). Unlike white-tailed eagles, golden eagles prefer small mammals, terrestrial birds, and carrion (Watson 2010). A recent study from Poland showed that mammals dominated the biomass of prey consumed by golden eagles in the Carpathian Mountains (68.4%), including roe deer (*Capreolus capreolus*, 33.2%), martens (*Martes* sp., 17%), red fox (*Vulpes vulpes*, 6.3%), European hare (*Lepus europaeus*, 4.3%), and avian prey (31.4%) including the common buzzard (*Buteo buteo*, 6.7%), Ural owl (*Strix uralensis*, 5%), domestic chicken (*Gallus gallus domesticus*, 3.7%), and common raven (*Corvus corax*, 3.6%) (Stój and Kruszyk 2021). The cadmium levels in terrestrial mammals could be higher than in terrestrial birds and waterfowl (Mochizuki et al. 2008). The liver and kidneys of roe deer, which are the dominant prey of golden eagles in this part of Europe (Stój and Kruszyk 2021), could contain several or dozens of mg kg^−1^ Cd (Finďo et al. 1993; Durkalec et al. 2015). The tissues of martens, which account for a significant fraction of the golden eagle’s prey (Stój and Kruszyk 2021), can contain considerable levels of Cd, ranging from 0.11 to 0.14 mg kg^−1^ ww in muscle tissue to almost 1 mg kg^−1^ in the liver (Alleva et al. 2006; Goretti et al. 2018). The interspecific differences in Cd content may also have been because all golden eagles came from one area located in the Carpathian Mountains, which has higher background Cd levels compared to other unpolluted areas of Poland. High and anomalous Cd levels in soils from this area can be linked to the geochemistry of the parent rock materials (Birke et al. 2017). Elevated Cd levels have also been reported in other species of animals from the Carpathian region. Recent studies on European bison (*Bison bonasus*) (Klich et al. 2021) and bank voles (*Myodes glareolus*) (Mikowska et al. 2014) were performed in similar areas in the Carpathian Mountains and showed higher hepatic Cd levels in these two herbivorous species than those found in other unpolluted regions of Poland. The levels of Cd in the liver of both eagle species found in this study were below the suggested toxicity threshold of 3 mg kg^−1^ and were comparable to those found by other authors (Fig. 4).

### Other trace elements

**Fig. 5:**
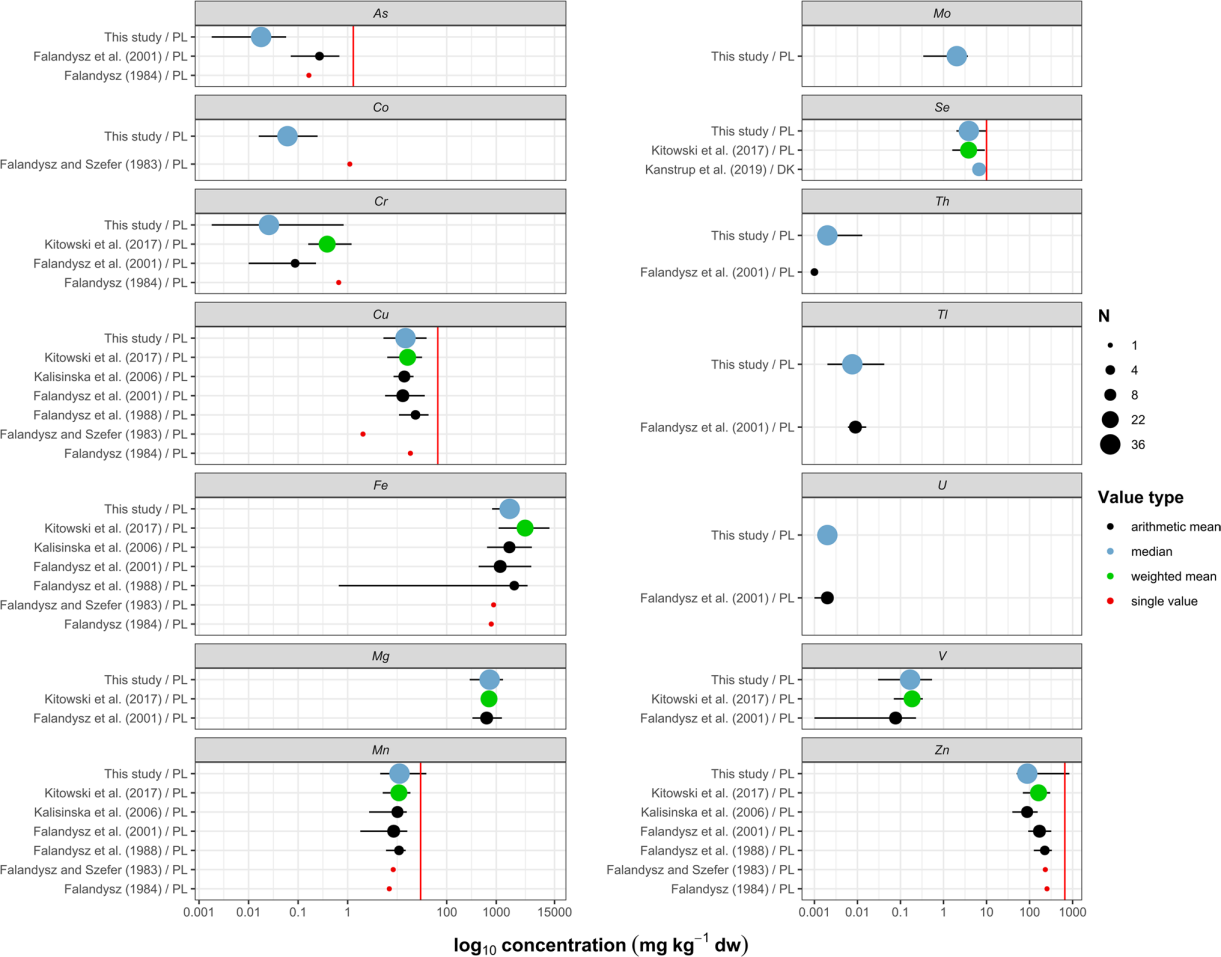
Concentrations of trace elements in the livers of white-tailed eagles (*Haliaeetus albicilla)*. Results are presented on a log_10_ scale (in mg kg^−1^ dw). Dots represent central value (mean or median), and whiskers show minimum and maximum. The dot size shows the number of birds tested (*N*). Gray indicates results that were converted from ww to dw. The red solid line represents literature thresholds for specific elements (in mg kg^−1^ dw): As, 1.3 (Camardese et al. 1990); Cu, 66 (Puls 1988); Mn, 29.7 (Puls 1988); Se, 10 (Eisler 1993); and Zn, 660 (Puls 1988). Countries are shown using a two-letter ISO 3166–1 alpha-2 code. (For interpretation of the references to color in this figure legend, the reader is referred to the web version of this article.) (Falandysz and Szefer 1983; Falandysz 1984; Falandysz et al. 2001; Kalisińska et al. 2006; Kitowski et al. 2017; Kanstrup et al. 2019)

The levels of As in the livers of the golden eagles and white-tailed eagles were generally low and ranged from < LOQ to 0.058 mg kg^−1^. Although the hepatic As in golden eagles seemed to be much lower than that found in white-tailed eagles (Supplementary Fig. S2), the difference was not confirmed by the GLM analysis (*Χ*^2^ = 1.21, *p* = 0.27). The levels we found in the eagles were comparable to those recently reported in other raptors from Slovakia (Hurníková et al. 2021) but tens of times lower than those found in previous decades in white-tailed eagles from the Baltic coast (Fig. 5). This difference is likely due to the high proportion of marine fish in the diet of white-tailed eagles from the coast (Falandysz et al. 1988). It was shown that marine fish could contain much more As than freshwater fish species (Mania et al. 2015), which may explain the higher levels of this element in white-tailed eagles reported previously from the Baltic coast region (Falandysz 1984; Falandysz et al. 2001). None of the tested individuals had hepatic As higher than the suggested threshold of 1.3 mg kg^−1^.

Cobalt is an essential element, and its concentration in the livers of birds shows low interspecific variation (Kim et al. 1998; Mansouri et al. 2012). The levels of Co we found in the livers of golden and white-tailed eagles were similar (Supplementary Table S3). Our results were more than 14 times lower than that reported from one specimen of white-tailed eagle found at the Baltic shore in the northwestern part of Poland in the early 80 s (Fig. 5) and comparable to those found in bald eagles (*Haliaetus leucocephalus*) from the Great Lakes (Nam et al. 2012).

Copper is a crucial micronutrient and component of many metalloenzymes, including those involved in respiration, protection against oxidative stress, immunity, growth and development, and proper nervous system functioning (Suttle 2010). Our data showed interspecific differences in the concentration of Cu (Supplementary Fig. S1). Hepatic Cu may vary among different bird species and can be challenging to interpret due to many factors, including the different sensitivities among species (Eisler 1993), age of the birds (Takekawa et al. 2002), season (Cohen et al. 2000), and body condition (Esselink et al. 1995; Jager et al. 1996). A recent review monograph by Łanocha-Arendarczyk and Kosik-Bogacka (2019) showed that the averaged liver Cu content estimated from literature data of raptor studies in Europe was about 17 mg kg^−1^ with slightly higher levels in predators of medium-sized birds and mammals compared to piscivorous birds of prey, which in line with the differences between both species that were confirmed in this study. The estimated marginal mean Cu in the livers of the golden eagles was 23.17 mg kg^−1^ and was more than 40% higher than in the white-tailed eagles (Wald’s *X*^2^ = 4.01, *p* = 0.045). The Cu analysis results in white-tailed eagles’ livers were consistent with previous findings in this species (Fig. 5) and suggest that these levels represent background concentrations.

Chromium is a naturally occurring element released into the environment from natural and anthropogenic sources (Prasad et al. 2021). It has different oxidation states, but two of them are biologically relevant: mutagenic and carcinogenic hexavalent, and trivalent, which has been previously thought to be essential for living organisms. However, its positive role has recently been questioned (Vincent and Lukaski 2018). Our results did not show species-specific, seasonal, or geographical patterns in the eagles’ hepatic Cr. The results of the total hepatic Cr reported here were within the range from < LOQ to 830 mg kg^−1^ and were much lower than reported by other authors (Fig. 5).

**Fig. 6:**
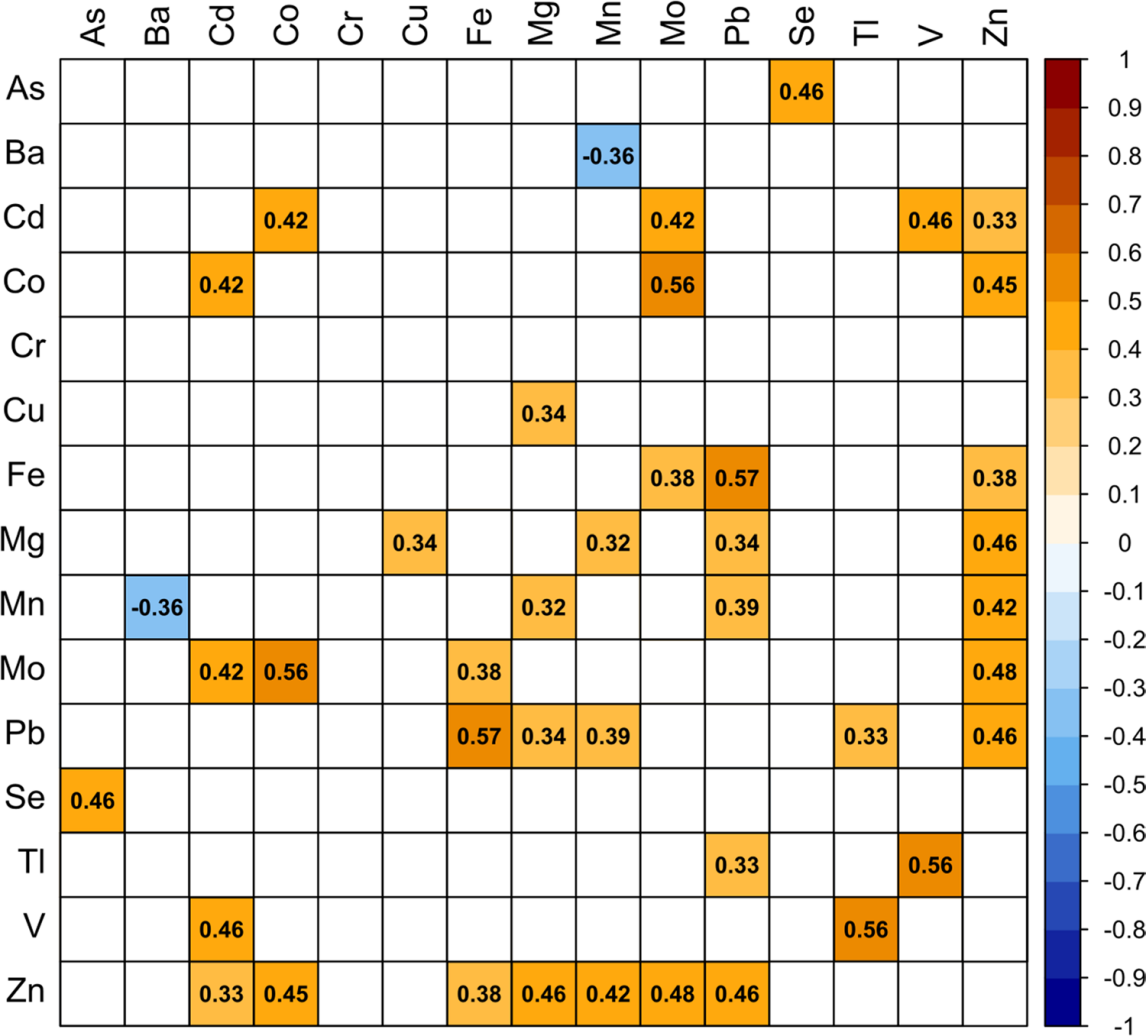
Spearman’s rank correlation coefficients (*ρ*) between metals and metalloids in the livers of white-tailed eagles (*Haliaeetus albicilla*). The squares’ color corresponds to the level of correlation: with 1 indicating a positive correlation (dark orange) and -1 indicating a negative correlation (dark blue). To facilitate the interpretation, numerical values of correlations are shown and non-significant correlations (*p* > 0.05) are left blank. (For interpretation of the references to color in this figure legend, the reader is referred to the web version of this article.)

Previous studies indicated that the Fe content in the livers of birds of prey ranged from 1030 to 2500 mg kg^−1^, which was consistent with the median Fe content in the livers of the golden eagles and white-tailed eagles found in this study (Supplementary Table S3). We found elevated Fe levels greater than 4000 mg kg^−1^ in two white-tailed eagles. The results of previous studies suggest that an increased Fe content in the liver may be related to an ongoing infection (Kalisińska et al. 2008) or accompanied by high Pb levels (Lewis et al. 2001). The latter point seems to correspond with our results, which showed a positive correlation between Fe and Pb (Fig. 6).

The GLM analysis revealed a difference in hepatic Mn between the golden and white-tailed eagles, in which the estimated marginal means were 22.38 and 13.90 mg kg, respectively (Wald’s *X*^2^ = 4.74, *p* = 0.029). A difference was also noted between the eagles from the northwestern part of Poland compared to the other regions (Supplementary Fig. S3; Wald’s *X*^2^ = 30.0, *p* = 1.38 10^−6^). The levels of Mn obtained in this study correspond with the results reported by other authors on white-tailed eagles (Fig. 5) and also common buzzards (9.18 mg kg^−1^) and common kestrels (14.3 mg kg^−1^) from Korea (Kim and Oh 2016), and different species of raptors from Poland (Kalisińska and Budis 2019).

Selenium is an essential metalloid trace element incorporated into many selenoproteins that have a wide range of crucial functions in organisms, including antioxidant and anti-inflammatory effects and thyroid hormone synthesis (Rayman 2012). Moreover, Se plays an essential role in protecting the organism from Hg poisoning by forming stable HgSe complexes that do not exhibit toxic effects (Yang et al. 2008; Burger and Gochfeld 2021). Although Se is essential for many organisms, it has a narrow tolerance spectrum and, in excess, may be toxic. Birds have been shown to have high sensitivity to Se (Chapman et al. 2010). In our study, all tested individuals had hepatic Se below 10 mg kg^−1^. Although the level of Se in the liver of the white-tailed eagles seemed to be almost twice as high as in the golden eagles (Supplementary Fig. S4), the difference was not confirmed by the GLM analysis (Wald’s *X*^2^ = 2.39, *p* = 0.12).

Thallium is one of the most toxic metals that have no role in living organisms. It is found in metal sulfide Pb–Zn deposits and is a common byproduct in the non-ferrous metal industry (Lis et al. 2003). High concentrations of Tl were found in plants (Wierzbicka et al. 2004), small mammals (Dmowski et al. 1998), and birds (Dmowski 2000) living in the area polluted by the Zn smelting industry in southern Poland. The median concentrations of Tl in the livers of the golden eagles and white-tailed eagles were 0.005 and 0.008 mg kg^−1^, respectively, and were similar to the levels found by Falandysz et al. (2001) in the livers of white-tailed eagles from northern Poland. The GLM analysis of our results showed site-specific differences in liver Tl (Wald’s *X*^2^ = 9.32, *p* = 0.025), with the highest levels in southwestern Poland which were twice as high as those found in the southeastern part of the country (Supplementary Fig. S4). This finding corroborates the results of Tl monitoring in topsoil which showed high Tl levels in the southwest and southcentral part of Poland (Salminen et al. 2005).

Zinc is an essential trace element commonly found in the organism as a component of numerous metalloenzymes playing roles in almost all signaling and metabolic pathways in living organisms. Both its deficiency and excess can lead to adverse health effects. The median hepatic Zn found in this study correspond with those reported previously from Polish white-tailed eagles (Fig. 5) and other raptor species reported elsewhere (Kosik-Bogacka and Łanocha-Arendarczyk 2019) and can be considered normal. Our results showed site-specific differences in hepatic Zn (Wald’s *X*^2^ = 19.23, *p* = 2.45 10^−4^) (Supplementary Fig. S4). We also found excessive Zn levels (843.7 and 691.2 mg kg^−1^) in two white-tailed eagles. These two birds came from areas of Poland without any industrial activity regarded as unpolluted. Although the levels we found in these two individuals may be considered toxic to poultry (Puls 1988) and indicate overexposure of birds to Zn, they likely cannot be regarded as a potential cause of the birds’ deaths. The direct cause of death of the first individual was poisoning by anticoagulant rodenticides (individual no. #34, (Sell et al. 2022). The second bird was found to have been exposed to anticoagulant rodenticides and carbofuran, which was considered the probable cause of death (individual no. #37, (Sell et al. 2022)).

### Correlations among elements

The Spearman’s rank correlation test showed a moderate negative correlation between Mn and Ba and 20 moderate positive correlations between the other elements studied, including As-Se, Cd-Zn, Mn-Pb, Pb-Tl, Pb–Zn, and Tl-V (Fig. 6). The moderate positive correlations between Zn and other metals, especially Cd, Co, Fe, and Pb, were consistent with previous findings, where positive correlations between Zn and other metals in the liver were reported in seabirds (Norheim 1987; Barrales et al. 2021), ducks (Lucia et al. 2008), white-tailed eagles (Kitowski et al. 2017), and other species (Kim et al. 2009). Correlations between these metals may be due to the shared affinity to metallothioneins (Sutherland and Stillman 2011) expressed in the liver and responsible for metal metabolism and detoxification (Nordberg and Nordberg 2009). The moderate positive correlation between As and Se in this study corroborates previous evidence (Lucia et al. 2012) and can be explained by their interaction (Sun et al. 2014). Selenium in small amounts has been shown to ameliorate As toxicity in different ways (Sah and Smits 2012).

### Temporal trends of Pb and Cd

**Fig. 7:**
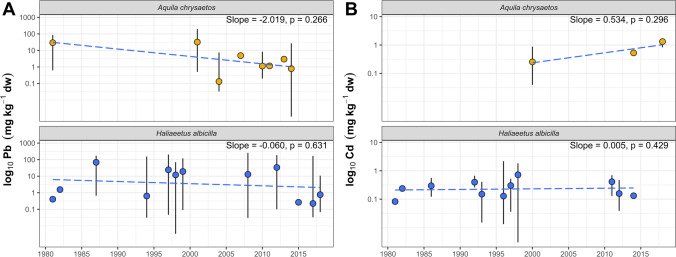
Temporal changes of Pb (**A**) and Cd (**B**) concentrations in the livers of golden eagles (*Aquila chrysaetos*) and white-tailed eagles (*Haliaeetus albicilla*). The dot and whisker plots represent central value and min–max range

The results obtained in this study and those extracted from articles identified by the systematic review were used to verify temporal trends in the eagles’ hepatic Pb and Cd levels. The concentrations of Pb and Cd in the livers of eagles over the last four decades are shown in Fig. 7. Although a slight decrease in Pb levels could be visually observed in the livers of golden eagles and white-tailed eagles (Fig. 7A), no trends were confirmed statistically (*p* > 0.05). This is in line with the results of previous work summarizing Pb levels in different raptor species across Europe, which did not reveal clear evidence of decreasing exposure of birds to this toxic element despite temporal changes being visible (Monclús et al. 2020). This could be due to differences in sample sizes, analytical methods, or spatial variation between the studies included in the analysis. In our work, we also did not find any temporal trends in hepatic Cd in either species (Fig. 7B).

## Conclusions

Our study provides information about the levels of Cd, Pb, and other metals and metalloids that were quantified in the livers of golden and white-tailed eagles that were found dead over the past two decades in Poland, and summarizes the results of previous work concerning hepatic levels of trace elements in these two species of raptors. We assessed the obtained hepatic levels by comparing them with reference values of elements for birds found in the literature. The results showed that the levels of most elements, excluding Mn, Pb, and Zn, were below the suggested levels associated with potential adverse health effects in birds. Although our results revealed that almost half of the tested eagles had Pb levels indicating past exposure to this toxic metal, the number of birds with hepatic Pb exceeding the suggested threshold of sublethal poisoning was much lower than reported previously in Poland and other countries. None of the birds tested in this study has hepatic Pb exceeding the level of clinical poisoning. Given the small number of available data on hepatic Pb levels in eagles, there was no clear evidence of its decline over the years. Further monitoring of this element in the tissues of eagles is needed to verify the effect of newly implemented restrictions on the use of lead in hunting.

## Supplementary Information

Below is the link to the electronic supplementary material.

Supplementary file1 (XLSX 62 KB)

Supplementary file2 (PDF 1013 KB)

## Data Availability

All data generated or analyzed in this work are within the article and its Supplementary Information files.
