# Supplementary material for: Lead, cadmium, and other trace elements in the liver of golden eagles and white-tailed eagles: recent data from Poland and a systematic review of previous studies
Source: Environ Sci Pollut Res Int. 2022 Dec 30;30(13):38566–81. doi: 10.1007/s11356-022-25024-y (PMC10039830; doi:10.1007/s11356-022-25024-y)
Supplement: Supplementary file 2 — Supplementary file2 (PDF 1013 KB) [file 11356_2022_25024_MOESM2_ESM.pdf]

Lead, cadmium, and other trace elements in the liver of golden eagles and white-tailed eagles: Recent data from Poland and a systematic review of previous studies

Maciej Durkalec[1,*], Agnieszka Nawrocka[1], Ignacy Kitowski[2], Aleksandra Filipek[1], Bartosz Sell[1], Mirosława Kmiecik[1], and Piotr Jedziniak[1]

[1]Department of Pharmacology and Toxicology, National Veterinary Research Institute, Aleja Partyzantów 57, 24-100 Puławy, Poland;

[2] Department of Zoology and Animal Ecology, University of Life Sciences in Lublin, Akademicka 13, 20-950, Lublin, Poland

***Corresponding author:** Maciej Durkalec, ORCID: 0000-0001-6036-7953
maciej.durkalec@piwet.pulawy.pl

Supplementary Table S1
Results of the analysis of DORM-4 (fish protein) certified reference material.

Element	LOQ (mg kg^{-1})	Measured value (mg kg^{-1})	Certified value (mg kg^{-1})	Recovery (%)
^{75}As	0.0009	6.4797	6.870	94.3
^{137}Ba	0.0009	-	-	-
^{9}Be	0.0011	-	-	-
^{111}Cd	0.0008	0.292	0.299	97.7
^{59}Co	0.0008	0.2221	0.250*	88.8
^{52}Cr	0.0009	1.739	0.0187	93.0
^{63}Cu	0.0015	13.728	15.700	87.4
^{56}Fe	0.0013	293.048	343.000	85.4
^{24}Mg	0.001	781.330	910.000	85.9
^{55}Mn	0.0011	2.764	3.170	87.2
^{95}Mo	0.015	0.2601	0.290*	89.7
^{208}Pb	0.0013	0.3812	0.404	94.3
^{80}Se	0.0011	0.3395	3.450	98.4
^{232}Th	0.001	-	-	-
^{205}Tl	0.001	-	-	-
^{238}U	0.001	0.0543	0.050	108.7
^{51}V	0.001	1.389	1.570	88.4
^{66}Zn	0.0025	44.245	51.600	85.7

*Informative value

Supplementary Table S2
Suggested thresholds for metals and metalloids in avian liver linked with specific health effects.

Element	Effect	Liver level (mg kg^{-1} d. w.)	Explanation	Reference
As	no effect/background	1.67	Based on observational study in wading birds	(Goede 1985)
	subclinical poisoning	1.3	Level associated with disturbances in normal activity patterns, growth, brain and liver biochemistry in mallards (*Anas platyrhynchos*) dietary exposed to sodium arsenate	(Camardese et al. 1990)
	elevated	6.6-33*	-	(Eisler 1988)
	poisoning	>33*		
Cd	no effect/bacground	<3	-	(Scheuhammer 1987)
	toxic	148.5–231*	Threshold effect level for hepatic, renal, or testicular toxicity	(Wayland and Scheuhammer 2011)
	high	16.5–66*	Suggested for poultry	(Puls 1988)
	toxic	49.5–660*		
Cu	adequate	9.9–49.5*	Suggested for chickens	(Puls 1988)
	toxic-chronic	66–495*		
	toxic-acute	907.5–2062.5*		
Cr	adequate	0.33–1.32	Suggested for poultry	(Puls 1988)
Mn	adequate	6.6–13.2*	Suggested for poultry	(Puls 1988)
	high	13.2–19.8*		
	toxic	>29.7*		
Pb	abnormal	>1	-	(Neumann 2009)
	subclinical poisoning	6.6-19.8*	-	(Franson and Pain 2011)
	clinical poisoning	19.8–33*	-	
	severe clinical poisoning	>33*	-	
Se	adequate	1.155–3.3*	Indicate appropriate nutritional status in poultry	(Puls 1988)
	high	6.6–19.8*	Indicate excessive Se but not considered as toxic for poultry	
	toxic	13.2–75.9*	Lower hatchability rate and teratogenic effects in poultry	
	no effect/background	<10	Freshwater and terrestrial birds	(Eisler 1993)
	potentially toxic	10–20	May indicate Se poisoning if present along with specific toxicity symptoms (Susceptibility may vary between species)	(Ohlendorf and Heinz 2011)
	toxic	20–25	Levels based on observational studies on mallards and could be used as diagnostic when accompanied with emaciation, bilateral alopecia of head and neck, maxillary nail necrosis, and hepatic lesions	(Ohlendorf and Heinz 2011)
	toxic	357–735	Levels associated with different tissue lesions	(Franson et al. 2007)
Zn	adequate	82.5–132*	Indicate appropriate nutritional status in poultry	(Puls 1988)
	high	297–990*	Indicate excessive Zn but not considered as toxic for poultry	
	toxic	660–2310*	Indicate toxicity in poultry	
	toxic	2100	-	(Eisler 1993)

*value converted from w.w. to d.w. using a factor of 3.3 (Helander et al. 2021)

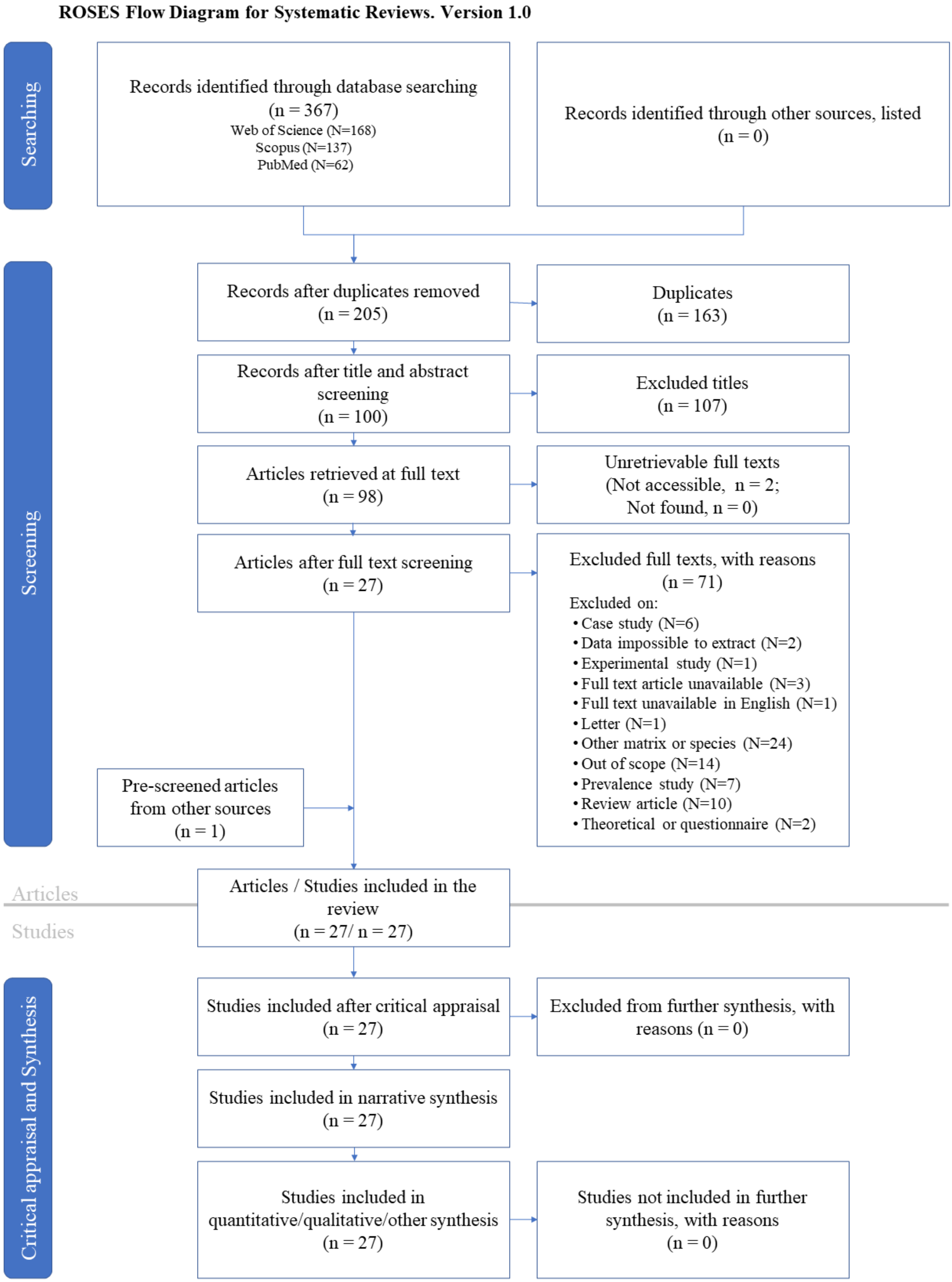

Supplementary Fig. S1
Flow diagram (according to (Haddaway et al. 2018))

Supplementary Table S3

Observed concentrations of trace elements in the liver of golden eagles and white-tailed eagles. Results shown as number of birds tested (N), number of left-censored data (LC), mean, standard deviation (SD), median, median absolute deviation (MAD), minimum (min), and maximum (max). Results are expressed in mg kg^{-1} d. w.

Element	Statistics	Species	
		golden eagle (*Aquila chrysaetos*)	white-tailed eagle (*Haliaaetus albicilla*)
As	N (LC)	3 (0)	36 (0)
	mean ± SD	0.005 ± 0.003	0.020 ± 0.013
	median ± MAD	0.005 ± 0.004	0.018 ± 0.012
	min–max	<LOQ—0.008	<LOQ—0.057
Ba	N (LC)	3 (0)	36 (0)
	mean ± SD	0.148 ± 0.162	0.092 ± 0.082
	median ± MAD	0.099 ± 0.124	0.062 ± 0.046
	min–max	0.016—0.328	0.012—0.376
Be	N (LC)	3 (2)	36 (32)
	mean ± SD	0.003 ± 0.001	0.002 ± 0.001
	median ± MAD	<LOQ	<LOQ
	min–max	<LOQ—0.005	<LOQ—0.007
Cd	N (LC)	3 (0)	36 (0)
	mean ± SD	1.200 ± 0.328	0.171 ± 0.101
	median ± MAD	1.322 ± 0.187	0.158 ± 0.080
	min–max	0.828—1.449	0.039—0.470
Co	N (LC)	3 (0)	36 (0)
	mean ± SD	0.072 ± 0.020	0.072 ± 0.050
	median ± MAD	0.078 ± 0.016	0.061 ± 0.037
	min–max	0.050—0.089	0.016—0.247
Cr	N (LC)	3 (0)	36 (0)
	mean ± SD	0.02 ± 0.01	0.058 ± 0.14
	median ± MAD	0.026 ± 0.001	0.019 ± 0.017
	min–max	0.009—0.026	0.002—0.83
Cu	N (LC)	3 (0)	36 (0)
	mean ± SD	23.41 ± 2.975	16.01 ± 6.571
	median ± MAD	23.54 ± 4.136	14.71 ± 4.923
	min–max	20.38—26.33	5.272—39.17
Fe	N (LC)	3 (0)	36 (0)
	mean ± SD	1470 ± 789.3	1988 ± 939.1
	median ± MAD	1360 ± 916.6	1863 ± 600.2
	min–max	741.7—2309	825.4—5225
Mg	N (LC)	3 (0)	36 (0)
	mean ± SD	809.6 ± 39.87	723.6 ± 180.2
	median ± MAD	805.6 ± 49.92	734.5 ± 122.4
	min–max	771.9—851.4	290.7—1369
Mn	N (LC)	3 (0)	36 (0)
	mean ± SD	16.70 ± 7.252	11.52 ± 6.390
	median ± MAD	14.31 ± 4.967	11.13 ± 4.762
	min–max	10.96—24.85	4.536—38.88
Mo	N (LC)	3 (0)	36 (0)
	mean ± SD	1.976 ± 0.044	2.110 ± 0.665
	median ± MAD	1.951 ± 0	2.037 ± 0.483
	min–max	1.951—2.026	0.339—3.691
Pb	N (LC)	3 (0)	36 (0)
	mean ± SD	2.011 ± 1.810	1.616 ± 2.083
	median ± MAD	1.133 ± 0.538	0.766 ± 1.227
	min–max	0.807—4.093	0.067—10.76
Se	N (LC)	3 (0)	36 (0)
	mean ± SD	2.871 ± 0.379	4.492 ± 2.071
	median ± MAD	2.861 ± 0.538	3.877 ± 1.227
	min–max	2.498—3.255	1.997—9.680

Supplementary Table S3
(continued)

Element	Statistics	Species golden eagle (*Aquila chrysaetos*)	white-tailed eagle (*Haliaaetus albicilla*)
Th	N (LC)	3 (2)	36 (33)
	mean ± SD	0.004 ± 0.003	0.002 ± 0.002
	median ± MAD	<LOQ	<LOQ
	min–max	<LOQ—0.007	<LOQ—0.013
Tl	N (LC)	3 (0)	36 (9)
	mean ± SD	0.016 ± 0.019	0.011 ± 0.01
	median ± MAD	0.005 ± 0.001	0.008 ± 0.008
	min–max	0.004—0.038	<LOQ—0.042
U	N (LC)	3 (3)	36 (36)
	mean ± SD	<LOQ	<LOQ
	median ± MAD	<LOQ	<LOQ
	min–max	<LOQ	<LOQ
V	N (LC)	3 (0)	36 (0)
	mean ± SD	0.169 ± 0.085	0.176 ± 0.105
	median ± MAD	0.204 ± 0.040	0.167 ± 0.113
	min–max	0.072—0.231	0.030—0.541
Zn	N (LC)	3 (0)	36 (0)
	mean ± SD	108.2 ± 9.660	135.1 ± 161.2
	median ± MAD	103.5 ± 2.583	88.44 ± 18.70
	min–max	101.7—119.3	50.03—843.7

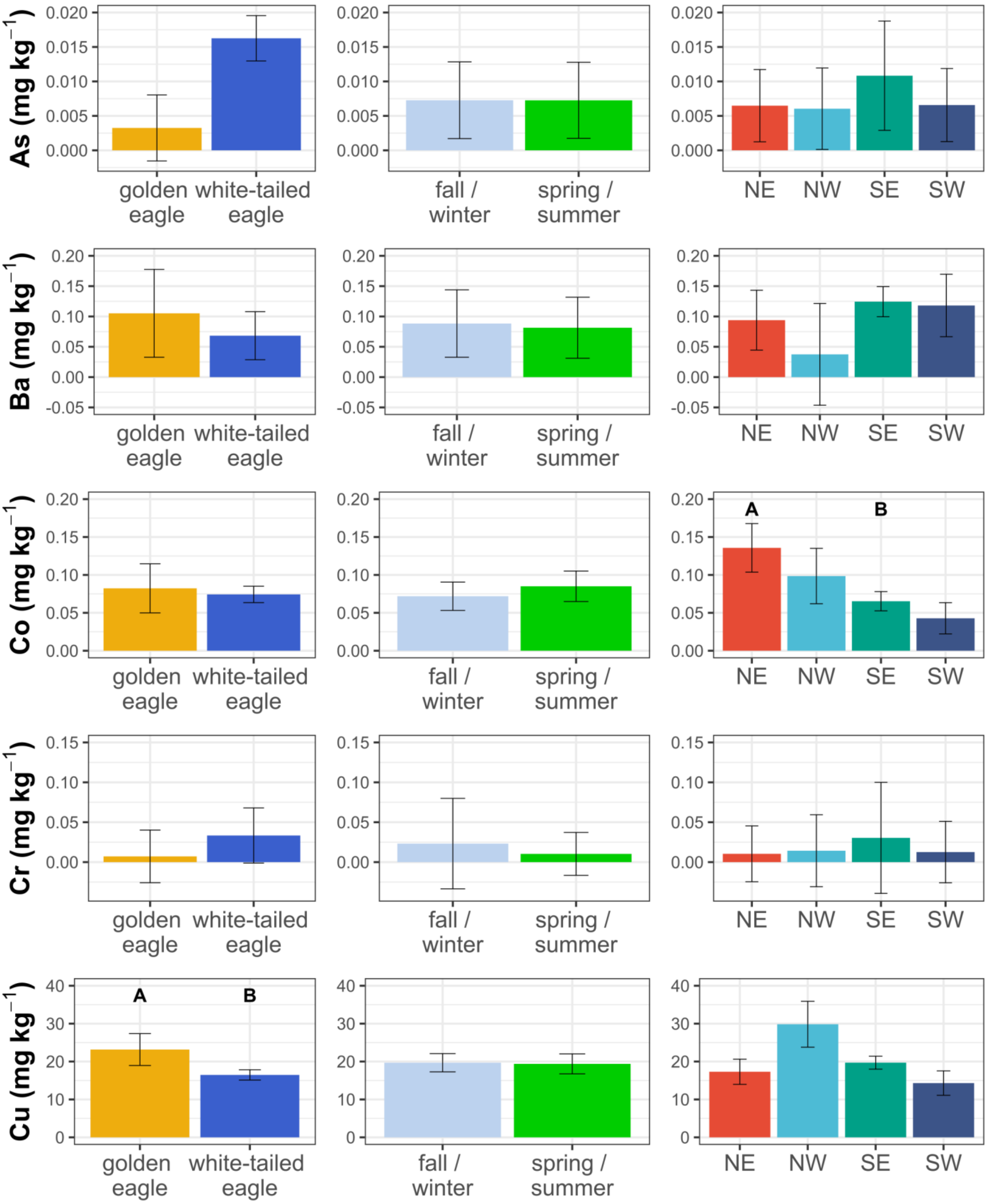

Supplementary Fig. S2
Differences in concentrations of As, Ba, Co, Cr, and Cu in the liver between golden eagles and white-tailed eagles, fall–winter and spring–summer seasons and among regions of Poland. Bar and whisker plots show estimated marginal means and standard errors that were computed from GLM models and back-transformed from the log scale. Differences between marginal means were verified on the log scale ($p \leq 0.05$).

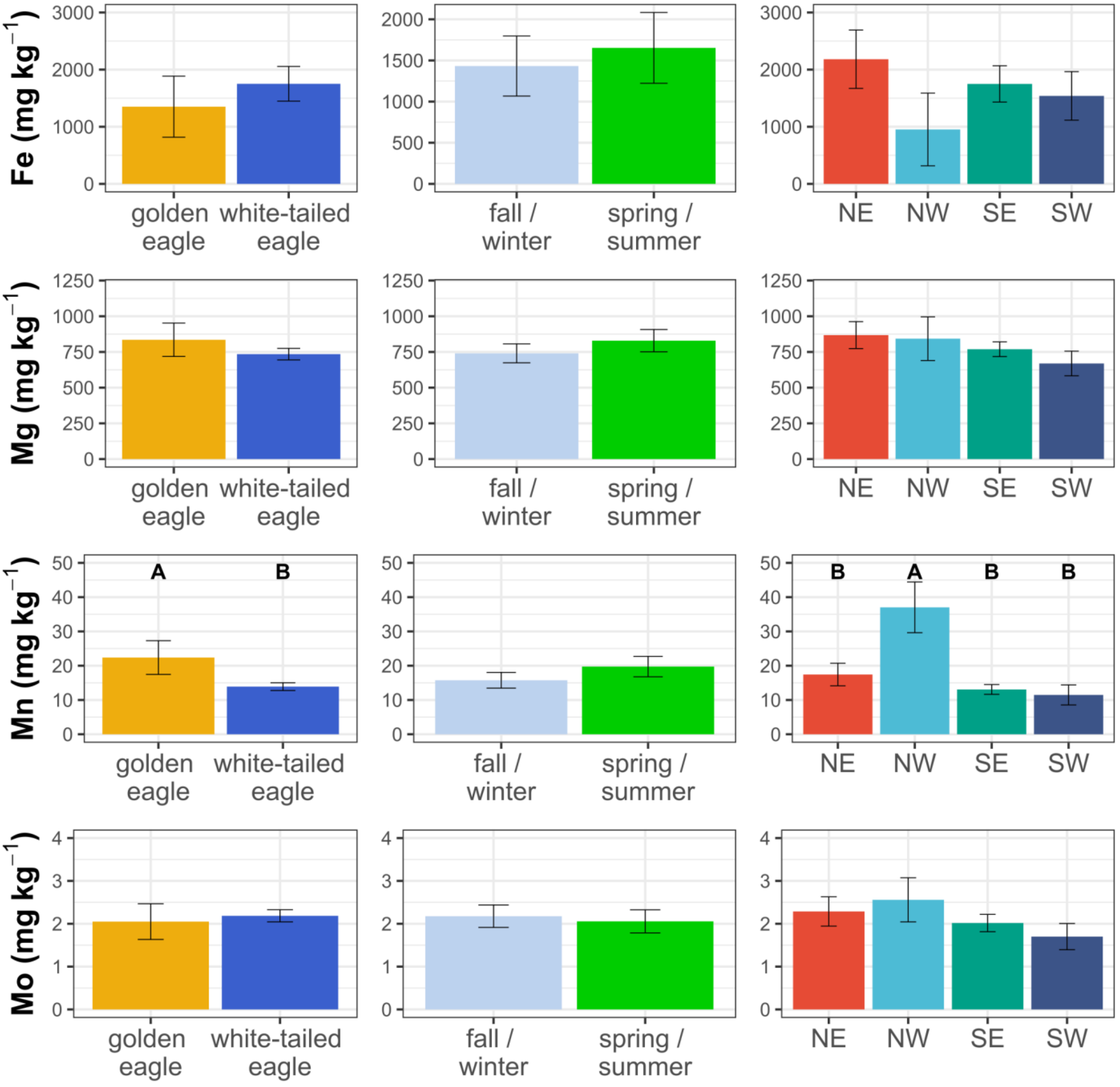

Supplementary Fig. S3
Differences in concentrations of Fe, Mg, Mn, and Mo in the liver between golden eagles and white-tailed eagles, fall–winter and spring–summer seasons and among regions of Poland. Bar and whisker plots show estimated marginal means and standard errors that were computed from GLM models and back-transformed from the log scale. Differences between marginal means were verified on the log scale ($p \leq 0.05$).

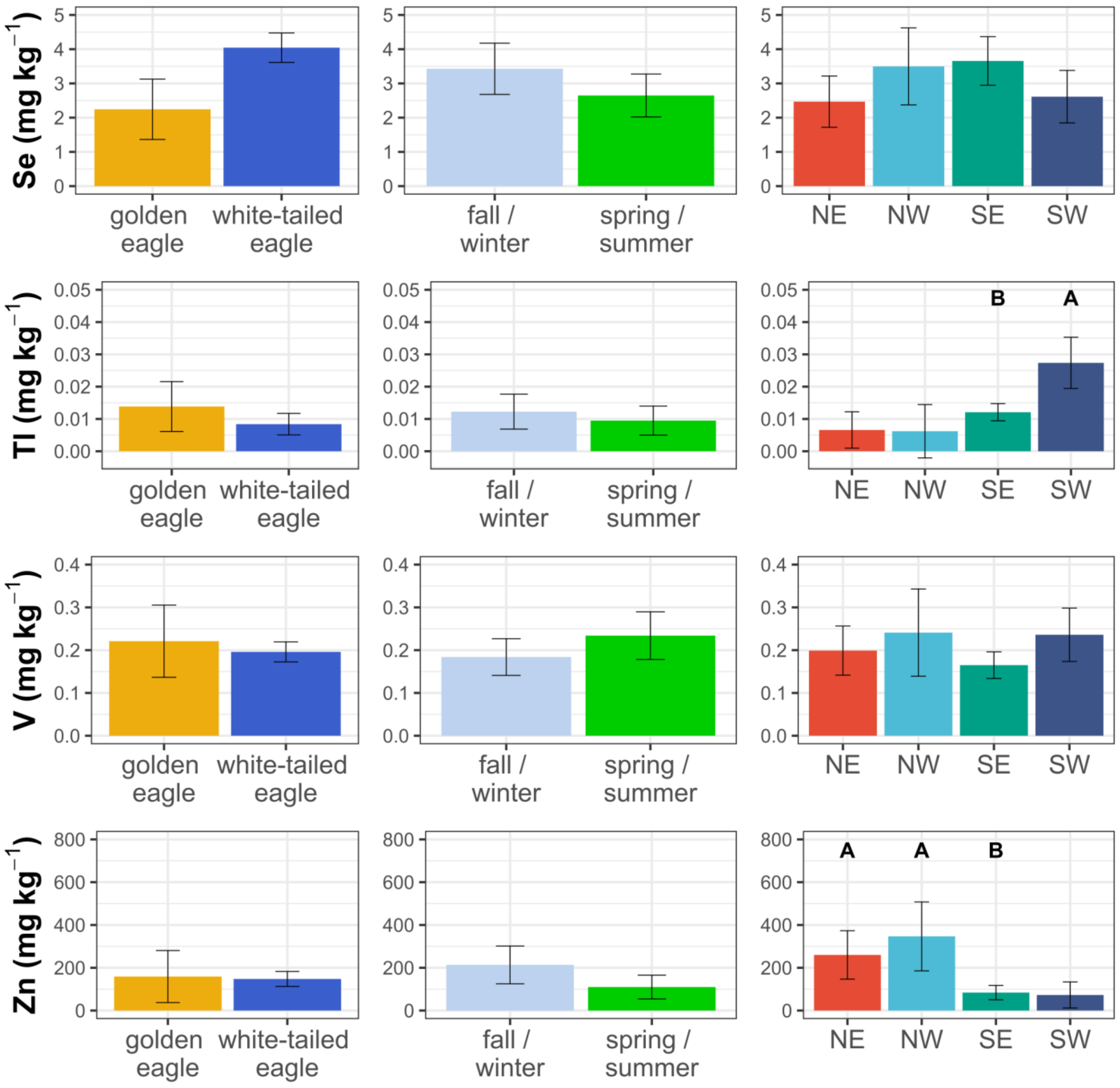

Supplementary Fig. S4
Differences in concentrations of Se, Tl, V, and Zn in the liver between golden eagles and white-tailed eagles, fall–winter and spring–summer seasons and among regions of Poland. Bar and whisker plots show estimated marginal means and standard errors that were computed from GLM models and back-transformed from the log scale. Differences between marginal means were verified on the log scale ($p \leq 0.05$).

References

Camardese MB, Hoffman DJ, LeCaptain LJ, Pendleton GW (1990) Effects of arsenate on growth and physiology in mallard ducklings. Environ Toxicol Chem 9:785–795. https://doi.org/10.1002/etc.5620090613

Eisler R (1988) Arsenic hazards to fish, wildlife, and invertabrates: a synoptic review. US Fish Wildl Serv Biol Rep 85:1–65

Eisler R (1993) Zinc hazards to fish, wildlife, and invertebrates: A synoptic review. Laurel, MD

Franson CJ, Pain DJ (2011) Lead in birds. In: Beyer WN, Meador JP (eds) Environmental Contaminants in Biota. Interpreting Tissue Concentrations, 2nd edn. CRC Press, Boca Raton, FL, pp 563–607

Franson JC, Hoffman DJ, Wells-Berlin A, et al (2007) Effects of dietary selenium on tissue concentrations, pathology, oxidative stress, and immune function in common eiders (Somateria mollissima). J Toxicol Environ Heal - Part A Curr Issues 70:861–874. https://doi.org/10.1080/15287390701212760

Goede AA (1985) Mercury, selenium, arsenic and zinc in waders from the Dutch Wadden Sea. Environ Pollut Ser A, Ecol Biol 37:287–309. https://doi.org/10.1016/0143-1471(85)90119-9

Haddaway NR, Macura B, Whaley P, Pullin AS (2018) ROSES Reporting standards for Systematic Evidence Syntheses: Pro forma, flow-diagram and descriptive summary of the plan and conduct of environmental systematic reviews and systematic maps. Environ Evid 7:4–11. https://doi.org/10.1186/s13750-018-0121-7

Helander B, Krone O, Räikkönen J, et al (2021) Major lead exposure from hunting ammunition in eagles from Sweden. Sci Total Environ 795:148799. https://doi.org/10.1016/j.scitotenv.2021.148799

Neumann K (2009) Bald eagle lead poisoning in winter. In: Watson RT, Fuller M, Pokras M, Hunt WG (eds) Ingestion of Lead from Spent Ammunition: Implications for Wildlife and Humans. The Peregrine Fund, Boise, Idaho, USA, pp 210–218

Ohlendorf HM, Heinz GH (2011) Selenium in birds. In: Beyer NW, Meador JP (eds) Environmental Contaminants in Biota. Interpreting Tissue Concentrations Interpreting Tissue Concentrations, 2nd edn. CRC Press, Boca Raton, FL, pp 669–701

Puls R (1988) Mineral levels in animal health. Diagnostic data. Sherpa International, Clearbrook

Scheuhammer AM (1987) The chronic toxicity of aluminium, cadmium, mercury, and lead in birds: A review. Environ Pollut 46:263–295. https://doi.org/10.1016/0269-7491(87)90173-4

Wayland M, Scheuhammer AM (2011) Cadmium in Birds. In: Beyer WN, Meador J (eds) Environmental contaminants in biota. Interpreting tissue concentrations, 2nd edn. CRC Press, Boca Raton, FL, pp 645–668
